# Anatomical Safety Area for Periarticular Analgesic Infiltration through the Posterior Capsule in Total Knee Arthroplasty: Radiological Study in Magnetic Resonance

**DOI:** 10.3390/jcm13072123

**Published:** 2024-04-06

**Authors:** Marta Mifsut-Aleixandre, Damián Mifsut, Eva María González-Soler, Arantxa Blasco-Serra, Alfonso Amador Valverde

**Affiliations:** Anatomy Department, Universitat de Valencia, 15 Av. Blasco Ibáñez, 46010 Valencia, Spain; marta_ma1998@hotmail.com (M.M.-A.); eva.m.gonzalez@uv.es (E.M.G.-S.); arantxa.blasco@uv.es (A.B.-S.); alfonso.a.valverde@uv.es (A.A.V.)

**Keywords:** periarticular analgesic infiltration, radiological study, anatomical safety area, knee prosthesis, magnetic resonance anatomy

## Abstract

**Background**: One of the main challenges of orthopedic surgery is adequate pain management after total knee arthroplasty. This work aimed to determine the anatomical safety area for infiltration through the posterior capsule of the knee in prosthetic surgery using Magnetic Resonance Imaging (MRI). **Methods**: A descriptive, observational, cross-sectional study was performed on 126 knee MRIs. The variables studied were age, sex, and distance between different neurovascular structures of the popliteal fossa (tibial nerve, common peroneal nerve, and vascular bundle). Data were analyzed for normality (Kolmogorov–Smirnov) and variance homogeneity (Levène). A value of *p* < 0.05 and a confidence interval of 9% were considered statistically significant for all comparisons. Student’s *t*-test was used to compare the means between independent samples. **Results**: We observed statistically significant differences between the sexes regarding EP–EPS (external plateau–external popliteal sciatic nerve (common peroneal)), EP–IPS (external plateau–internal popliteal sciatic nerve (tibial)), and IP–PA (internal plateau–popliteal artery) measurements. The average distance between both nerves, EPS–IPS (external popliteal sciatic nerve and internal popliteal sciatic nerve), was 25.96 mm in females, while the value obtained in males was 29.93 mm, but this difference was not statistically significant. **Conclusions**: The average distance from the posterior capsule to the EPS and IPS nerves is greater in males than in females, despite no statistical differences. The presence of a lateralized arteriovenous bundle reduces the infiltration area of the external compartment. Regarding the safety area, infiltration of the internal compartment is safe since the volume diffuses into the muscle mass of the internal gastrocnemius upon injection. To infiltrate the external compartment, the needle must move at least 2 cm from the midline toward the external side (to exceed the maximum displacement of the neurovascular bundle established at 1.82 cm), and not advance beyond 0.76 cm (minimum distance at which we located the common peroneal nerve in the external compartment).

## 1. Introduction

Joint replacement surgery or total knee arthroplasty (TKA) is a clinically effective and cost-effective intervention with high success rates in terms of pain relief and functional capacity optimization in patients with advanced knee osteoarthritis [1]. TKA is associated with a significant pain component during the postoperative period [2], so approximately 30% and 60% of patients experience severe pain and moderate pain, respectively. To minimize severe postoperative pain in prosthetic knee surgery, different drugs and techniques have been used, including non-steroidal anti-inflammatories, opioids, and anesthesia techniques or nerve blocks. One of the main challenges in orthopedic surgery is adequate pain management after intervention and ensuring that mobilization, ambulation, and rehabilitation are performed as early as possible. In this way, an optimal and early analgesic control strategy after TKA guarantees favorable clinical and functional results, as well as shorter hospital stays and lower associated costs [2,3].

Peripheral nerve blocks are an excellent technique in the perioperative analgesia of total knee arthroplasty. Different nerves have been used as targets for a single or combined block, in a single injection, or continuous infusion. The first femoral nerve block has been implicated in controlling pain related to anterior thigh and medial lower leg pathology. The use of these blocks has resulted in decreased opioid use, earlier functional recovery, less pain, greater knee flexion, and shorter hospital stays. There are two modes of administration: single injection or continuous injection with an indwelling catheter. When administered as a single injection, analgesia usually lasts about 12–24 h, although residual effects may persist up to 48 h.

Regarding the obturator nerve, one portion penetrates the intra-articular space and innervates the knee joint; therefore, its adequate blockage can prevent pain in the popliteal area. Since the obturator nerve block is inconsistent with the isolated femoral nerve block, a variation called ‘3-in-1 block’ was developed, which blocks both the femoral nerve and the obturator and lateral femoral cutaneous nerves with a single anesthetic injection.

Adductor canal nerve block: The adductor canal is an aponeurotic tunnel in the middle third of the thigh. The femoral artery, the femoral vein, the vastus medialis nerve, and the saphenous nerve, the largest cutaneous branch of the femoral nerve, pass through the adductor canal. Ultrasound-guided adductor canal block provides a reliable method of blocking the saphenous nerve, which comprises the terminal sensory branch of the femoral nerve. One advantage is that the sensory block does not affect quadriceps motor function. Despite theoretical benefits such as decreased falls, analgesia in the posterior region of the knee may be insufficient.

For patients undergoing knee surgery, peri-articular/intra-articular infiltration analgesia is an analgesic technique comprising peri- and intra-articular injection and infusion of analgesics into surgical sites. In 2008, Kerr and Kohan introduced intraoperative periarticular analgesic injection (IPA) around the soft tissues of replaced knee joints, which involves the injection of local anesthetic drugs (bupivacaine, levobupivacaine, and/or ropivacaine) into affected tissues in TKA, as well as adjuvants such as non-steroidal anti-inflammatory drugs, corticosteroids, adrenaline, and morphine sulfate, which can increase the technique’s effectiveness and prolong the duration of its effect [4]. For an adequate distribution of drugs, the most important part of the technique lies in infiltrating the posterior region through the joint capsule, both in the medial and lateral compartments. In this way, good analgesia is achieved in the knee. However, this gesture carries a risk of injury to the vascular–nervous structures in the popliteal fossa. For this reason, many surgeons avoid infiltrating this anatomical region, causing the technique itself to fail since it does not provide sufficient analgesia.

The main objective of this study was to determine the anatomical area of safety for infiltration through the posterior capsule of the knee in prosthetic surgery using Magnetic Resonance Imaging (MRI).

## 2. Methods

### 2.1. Participants

A radiological study with 126 magnetic resonance images (MRI) was conducted. Images were obtained from 126 patients of our hospital center, who were studied during the differential diagnosis process for knee pain in routine clinical practice, thus avoiding additional economic costs.

MRI selection was limited to dates ranging from September to December 2021, with cases being chosen consecutively.

### 2.2. Inclusion Criteria

Inclusion criteria were:

- Age between 18 and 75 years old;
- Subjects with no trauma history or previous knee surgery, osteoarthritis, malformation, or tumors (including Baker’s cyst), which could alter the normal anatomy of the popliteal fossa;
- Sufficient image quality to perform measurements.

### 2.3. Exclusion Criteria

Exclusion criteria were:

- Age under 18 years old or over 75 years old;
- Subjects with trauma history or previous knee surgery, osteoarthritis, malformation or tumors (including Baker’s cyst), which could alter the normal anatomy of the popliteal fossa;
- Insufficient image quality to perform measurements.

### 2.4. Ethics

This study was in accordance with the Ethics Committee of the University Clinical Hospital and received the approval of the Committee of the Institute of Health Research.

To randomize the study and comply with Spanish Organic Law 3/2018 of 5 December on the Protection of Personal Data and Guarantee of Digital Rights and 41/2002 Regulating Basic Law of Patient Autonomy and Rights and Obligations, valid cases were coded to pseudonymize the radiological study. Similarly, images of selected cases were stored in Digital Imaging and Communication in Medicine formats on a password-protected disk and analyzed without the presence of case identification data on the screen.

### 2.5. MRI Analysis

MRA images were obtained with a 1.5 T system apparatus magnetic resonance system (SIEMENS HEALTHINEERS M-SOLA, Malverne, NY, USA) and a dedicated knee array. All subjects underwent imaging in the supine position. For our study, we used axial sequence, proton density, and fat saturation.

Acquisition voxel size: 0.56 × 0.39 × 3.00 mm^3^. Reconstruction voxel size: 0.39 × 0.39 × 3.00 mm^3^.

All measurements were performed by a single investigator, an orthopedic surgeon with over 25 years of experience, on a picture archiving and communication system (PACS) with a mouse pointer (cursor) and automated computer-processed calculations of lengths and angles. Linear measurements were recorded in increments up to 0.01 mm units.

**Figure 1:**
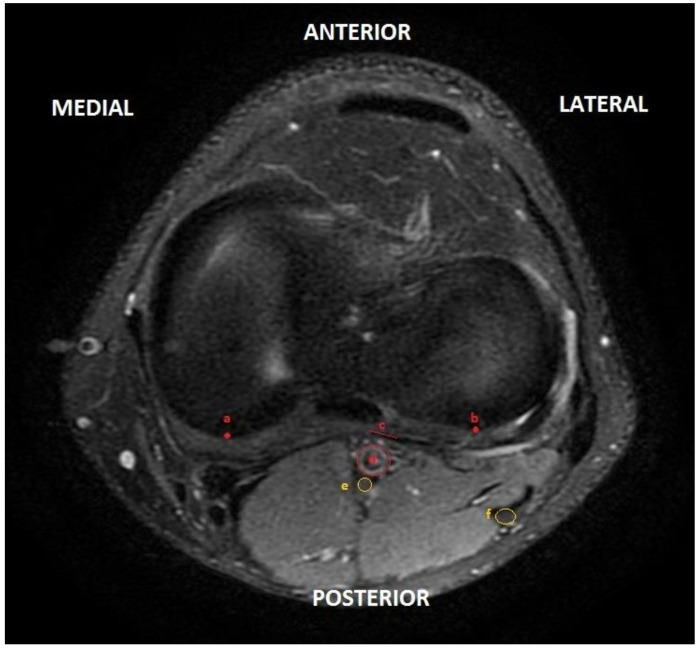
Axial section of the left knee in magnetic resonance imaging, with marks of the main elements: (**a**) center of the internal tibial plateau at the posterior level; (**b**) center of the external tibial plateau at the posterior level; (**c**) posterior capsule next to the popliteal artery; (**d**) popliteal artery; (**e**) internal popliteal sciatic nerve (tibial); (**f**) external popliteal sciatic nerve (common peroneal).

Axial cuts were created as reference points where the femoral condyles were no longer visible and the menisci were visible. These points coincided with the joint line and represented the puncture level of the posterior capsule. For the radiological analysis, we used Universal Viewer Zero Footprint Client 6.0 SP7. The study was performed using axial slices of MRI at the level of the joint line, as shown in Figure 1. The study area does not present any pressure deformity at this level as it coincides with the popliteal fossa; therefore, the anatomy does not exhibit distortion.

**Figure 2:**
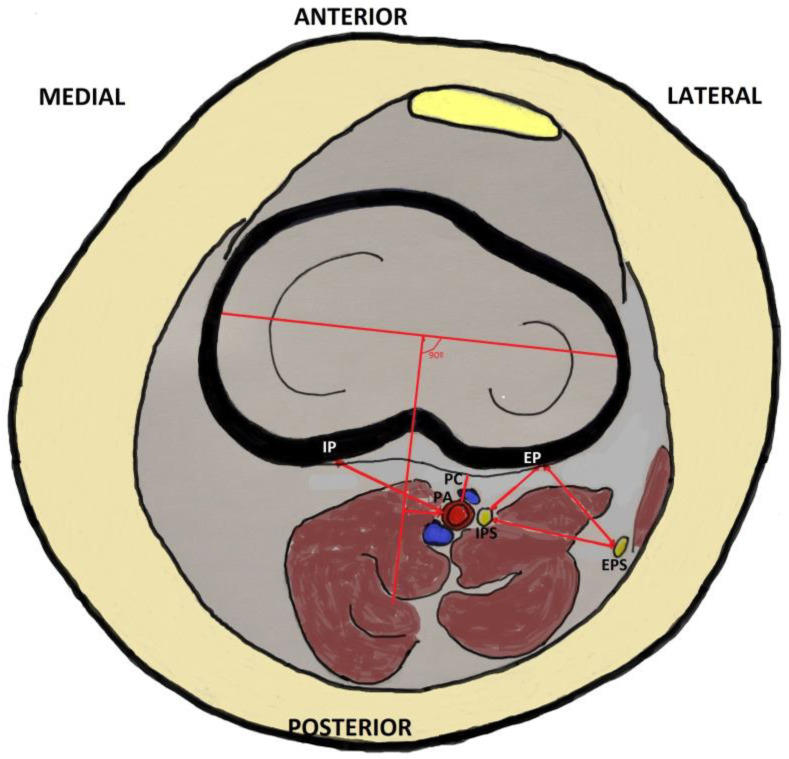
Graphic representation of the study area of the knee: IP = internal plateau, EP = external plateau, PC = posterior capsule, PA = popliteal artery, EPS = external popliteal sciatic nerve (common peroneal), IPS = internal popliteal sciatic nerve (tibial).

**Figure 3:**
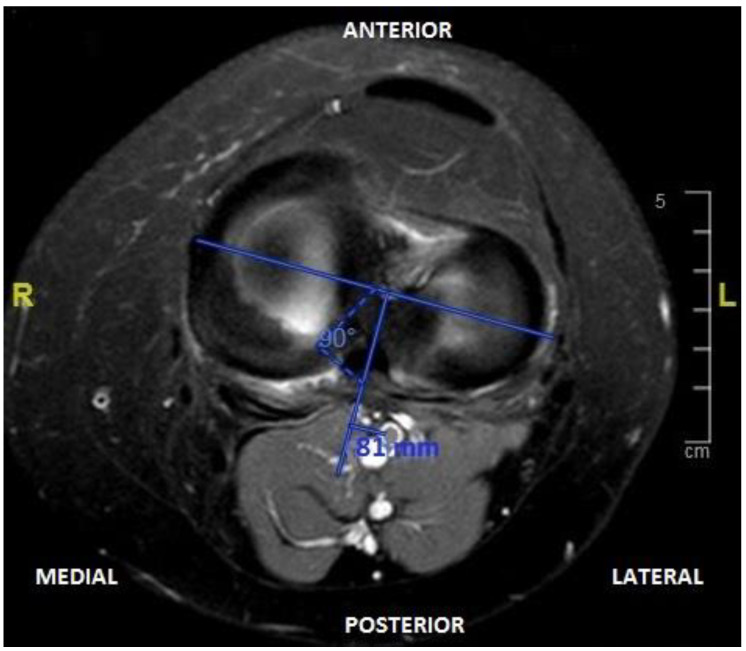
Axial section of the left knee in magnetic resonance imaging, where we observe the determination of the midline drawn from the center of the tibial spine at 90° from the transverse axis of the knee and distance from the popliteal artery to said line (IM–PA).

To determine the anatomical area of safety in analgesic periarticular infiltration through the posterior capsule, the following measures were conducted (Figure 2):

- Distance from the center of the posterior edge of the external tibial plateau to the common peroneal or external popliteal sciatic nerve (EP–EPS);
- Distance from the center of the posterior edge of the external tibial plateau to the internal popliteal sciatic nerve (EP–IPS);
- Distance between common and tibial peroneal nerve (external and internal popliteal sciatic nerve) (EPS–IPS);
- Distance from central posterior capsule to popliteal artery (PC–PA);
- Distance from the center of the posterior border of the internal tibial plateau to the popliteal artery (IP–PA).
- Distance from the intercondylar midline to the center of the popliteal artery (IM–PA). To perform this measurement, the line perpendicular to the coronal axis of both tibial plateaus was drawn at 90° and the distance from the center of the popliteal artery to this line was measured (Figure 3).

Measurements were determined from axial sections in millimeters.

### 2.6. Statistical Methods

To determine the sample size, a pilot study was conducted with 20 patients (10 men and 10 women) observing a standard deviation for the distance between both nerves of 2.8. Considering that the groups for both sexes have the same n (n1 = n2), differences in millimeters were estimated to be correct within 1 mm (error) with a probability of 0.95 Z (95%) = 1.96; n1 = n2 = 60.23. A total number of 122 cases is estimated.

Data were analyzed and represented on graphs using SPSS v.28 (IBM, New York, USA). They were expressed as the mean ± standard error mean (SEM) and analyzed for normality (Kolmogorov–Smirnov) and variance homogeneity (Levène). A value of *p* < 0.05 and a confidence interval of 9% were considered statistically significant for all comparisons. The means of independent samples were compared by Student’s *t*-test, in case of a normal distribution, or the non-parametric Mann–Whitney test, if the applicability conditions of the *t*-test were not met.

## 3. Results

A total of 126 cases were studied, 54 females and 72 males, aged between 20 and 76 years, with a mean age of 50.41 years (median 53 years). A total of 66 right knees and 60 left knees were analyzed. The sample presented a normal distribution, so parametric tests were applied. Table 1 shows the results of the measurements in our work.

**Figure 4:**
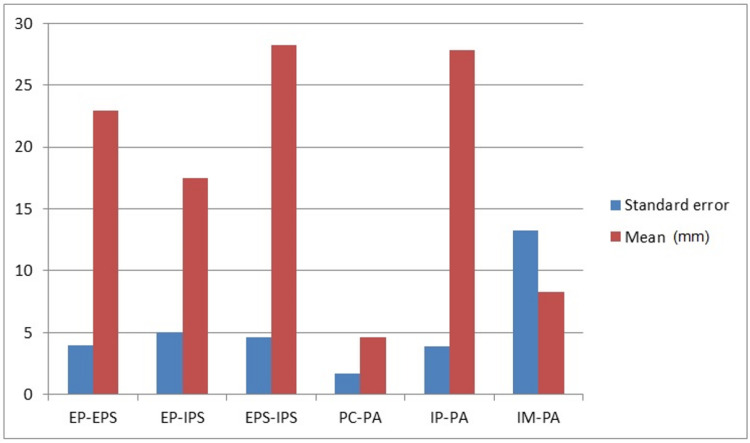
Mean and standard error for each of the measurements obtained.

For the inferential statistical analysis, Student’s *t*-tests for independent samples were performed given that the sample was normal and homogeneous. Statistically significant differences were observed in EP–EPS (t = 3.3497681094478; *p* = 0.00107424497595367). EP–IPS (t = −3.32948168511137; *p* = 0.00114614959079223), and IP–PA (t = −3.0752645551169; *p* = 0.00258772652965264). Measurements obtained for the female samples showed lower values than those obtained for the male samples. No statistical differences were found between the two knees for any value studied. No differences were found for the EPS–IPS (t = −5.26610324997478; *p* = 5.9502446151122), PC–PA (t = −1.87058100887924; *p* = 0.0637605973350425), and IM–PA (t = 0.35543128370598. *p* = 0.722870407791877) measures. The results are shown graphically in Figure 4.

## 4. Discussion

Total knee arthroplasty is commonly performed on patients with end-stage osteoarthritis to improve quality of life. Postoperative TKA procedures are followed by moderate to severe postoperative pain. The current ‘gold standard’ of treatment to relieve pain after knee surgery is epidural anesthesia or a continuous femoral nerve block [5,6]. Different adverse effects can result from these treatments, such as urinary retention, nausea, partial motor block, etc. [7,8]. In addition, postoperative pain in TKA limits early ambulation and range of motion, increasing the risk of thromboembolism and delaying rehabilitation, which decreases patient satisfaction and overall outcomes [9,10].

Multimodal analgesia in TKA improves patients’ functional recovery by maintaining normal muscle tone [11]. Peri-/intra-articular infiltration analgesia in TKA is an analgesic technique comprising peri- and intra-articular injection and infusion of analgesics in the surgical area. These two methods both use local anesthetics or possible adjuncts to block nociceptive impulses by inhibiting voltage-gated Na+ channels, albeit at different sites (neuronal terminal for local wound infiltration, peripheral nerve or central nervous system for nerve block); however, both exert analgesic effects. Peripheral nerve block and peri-articular/intra-articular infiltration analgesia are both recommended by the American Association of Hip and Knee Surgeons in post-operative analgesic guidelines.

In 2008, Kerr and Kohan introduced intraoperative periarticular analgesic injection (IPA) around the soft tissues of replaced knee joints, which involves the injection of local anesthetic drugs immediately after surgery [4]. This technique has demonstrated improvement in postoperative pain, with decreased pain scores, reduced hospital stays, reduced use of morphine drugs during the postoperative period, and an increase in postoperative mobilization [4,9,11,12,13].

Other local injection techniques around the knee have also been described, with different combinations of drugs and different doses. These techniques aim to achieve early functional rehabilitation and prevent immediate postoperative falls [11,12,14,15,16]. However, periarticular/intraarticular infiltration analgesia may affect wound healing and joint drainage, depending on the types of analgesics, injection sites (post-articular, periarticular, or intra-articular injections of analgesic medications), and injections. Single or continuous infusions may have different durations of analgesic efficacy.

Despite the literature support for LIA use, the best location for injecting analgesic cocktails (intra-articular versus peri-articular, versus combined intra-articular and periarticular) is still debated.

These publications on IPA techniques describe the anatomical points of infiltration: the periosteum, Hoffa’s fat, the popliteal area through the medial and lateral compartments, tissues around the arthrotomy, etc., as well as the total volume of fluid injected around the knee joint [4,11,17]. However, anatomical studies that specifically analyze the spread of the local anesthetic or its relationship to the anatomy and innervation of the knee, are scarce. To date, we have not found any study on the safety area for infiltration through the posterior capsule.

For the orthopedic surgeon, puncture and infiltration of the popliteal fossa from the anterior to posterior side, especially in the external compartment, causes some uncertainty due to the risk of infiltrating the popliteal neurovascular bundle or injuring the common peroneal nerve. Consequently, infiltration in this area is avoided, scarce, or insufficient due to ignorance or anatomical insecurity. Better knowledge of the distance between the posterior capsule and the common peroneal nerve, the distance between the common peroneal nerve and the posterior tibial nerve, and the distance between the posterior capsule and the neurovascular bundle will provide the surgeon with more confidence and security in this anatomical region.

In an anatomical study on six cadaver knees, Quinn’s team found that the distribution of the injected volume within the popliteal fossa was uneven, with less dye distribution in areas farther from the popliteal fossa than those proximal to it. The injections used with this IPA technique reached nerves that innervate the knee joint, causing potential motor block [18].

Although knee innervation varies greatly between people, it fundamentally consists of nerve branches related to the femoral, obturator, and sciatic nerves [19]. Innervation is differentiated into two main groups: anterior and posterior [19,20]. Genicular branches derive from both main groups that innervate the entire knee joint. The number of genicular nerves, as well as their distribution or anatomical arrangement, can vary greatly. They travel through different areas before entering the capsule and follow the same route as vascularization once inside the knee [19,21]. In Quinn et al.’s study, the distribution of the dye in the popliteal fossa was extensive, reaching the motor nerves, the tibial nerve, and the peroneal branches of the sciatic nerve. Due to the dye’s extensive distribution on the anterior surface of the femur at a distal level, the genicular branches of the femoral nerve were probably reached. However, these genicular branches were not dissected in the study. In our opinion, the common peroneal nerve can be blocked in this case, causing temporary paralysis and preventing early rehabilitation. Based on their findings, they felt that the current technique could be refined to better maximize local anesthetic delivery. The infiltrations were not adequately distributed throughout the distal popliteal fossa and did not reach the popliteus muscle sufficiently in its entire extension. For these reasons, they have considered improving and perfecting the technique using other different injection points, specifically directed at the distal popliteal fossa.

Tubtim et al. studied the distribution of the infiltrated volume through the medial posterior capsule in a study with 10 cadaver knees. They assessed whether there was diffusion from the medial to the lateral compartment to avoid puncturing the compartment. However, they observed that the injected volume in both groups, namely five knees in which 25 mL was injected and another five knees in which 50 mL was injected, did not cross the midline into the lateral compartment. Conversely, it was distributed proximally and distally through the medial compartment, with greater distance in the second group (50 mL) [22]. They concluded that infiltrating both compartments is necessary to achieve good analgesia. More studies are needed to establish the total volume required for good analgesia. Furthermore, the most appropriate injection points must be determined without producing motor blockades, which impede early rehabilitation.

To improve the technique and provide surgeons with security when infiltrating the posterior capsule of the knee, we conducted this anatomical study based on radiological resonance images.

For all the reasons mentioned, the external compartment presents a greater risk of infiltration; however, there is a fairly wide safety window at around two centimeters. Our study found statistically significant differences between both sexes in measuring distances between the posterior edge of the external plateau and the external and internal popliteal sciatic nerves (particularly the peroneus and tibial nerves), which were greater in men than in women. Although the distance between nerves was greater in men, these differences were not significant.

At the central–lateral level, the arteriovenous bundle is close to the posterior capsule. So, in addition to separating ourselves from the midline, we should not advance the puncture needle after crossing the posterior capsule. Although the distances between the posterior capsule and popliteal artery and from the midline to the popliteal artery were lower in women, they were not statistically significant. In the medial compartment, the space between the center of the internal plateau and the popliteal artery was more than 26.59 mm in women and 28.68 mm in men; therefore, there is a wide area of safety in the entire medial compartment.

In the lateral compartment, the arteriovenous bundle moves to the lateral side from the midline, closing the space for infiltration (mean translation of 8.3 mm) in both men and women, as previously described. We must enter the external compartment laterally, away from the vascular bundle, and without deepening or progressing the needle after passing through the capsule, to avoid reaching the EPS, which could block the nerve. In the medial compartment, a centralized infiltration in the compartment will not entail any risk.

There is little standardization in the literature regarding therapeutic regimens for periarticular infiltration. Multiple studies have compared different drug combinations, but with no precise definition of the ideal mixture. More research is needed on the pharmacology of infiltration to allow surgeons to choose what they prefer, as well as standardize volume and optimal infiltration points.

## 5. Conclusions

In conclusion, there is sufficient space between both the EPS and IPS nerves (tibialis posterior and common peroneus) to perform infiltration in the medial compartment, with no statistical differences between the sexes. Similarly, the presence of the lateralized arteriovenous bundle toward the external side reduces the infiltration space of the external compartment. We also observed a better safety area in the medial compartment than in the external one. Therefore, the risk of injuring some anatomical structures is greater with needle advancement, especially in the external compartment, which reaches the external popliteal sciatic nerve. Regarding the safety area, infiltration of the internal compartment is safe since the volume diffuses into the muscle mass of the internal gastrocnemius upon injection. To infiltrate the external compartment, it is necessary to move at least 2 cm from the midline toward the external side (to exceed the maximum displacement of the neurovascular bundle established at 1.82 cm) and not advance the needle beyond 0.76 cm (minimum distance at which the common peroneal nerve in the external compartment was located).

## Figures and Tables

**Table 1:** Measurements obtained in the study.

Measurement	Mean	Minimum	Maximum	Standard Error
EP-EPS distance	22.90 mm	7.6 mm	31.0 mm	3.92
EP-IPS distance	17.46 mm	8.5 mm	34.8 mm	4.99
EPS-IPS distance	28.23 mm	12.1 mm	39.9 mm	4.61
PC-PA distance	4.59 mm	1.5 mm	10.3 mm	1.64
IP-PA distance	27.79 mm	15.3 mm	37.9 mm	3.9
IM-PA distance	8.3 mm	1.6 mm	18.2 mm	13.25

EP: External tibial plateau; EPS: External popliteal sciatic nerve; IPS: Internal popliteal sciatic nerve; PC: Posterior capsule; PA: Popliteal artery; IP: Internal tibial plateau; IM: Intercondylar midline.

## Data Availability

No new data were created or analyzed in this study. Data sharing is not applicable to this article.

## Relevance Statement

The article provides greater security to the surgeon to infiltrate the popliteal fossa, through the posterior capsule in knee arthroplasty surgery, to improve post-surgical pain.

## Key Points

The space between IPS–EPS nerves is safe enough for infiltration. There is greater danger in the posterolateral compartment of the knee. Infiltration is possible after passing through the capsule without advancing the needle further.

## Abbreviations

MRI	Magnetic resonance imaging
EP	External tibial plateau
EPS	External popliteal sciatic nerve
IPS	Internal popliteal sciatic nerve
PC	Posterior capsule
PA	Popliteal artery
IP	Internal tibial plateau
IM	Intercondylar midline
TKA	Total knee arthroplasty
IPA	Intraoperative periarticular analgesic
SEM	Standard error mean
